# Health workforce issues for podiatry

**DOI:** 10.1186/1757-1146-4-S1-I2

**Published:** 2011-05-20

**Authors:** Peter M Brooks

**Affiliations:** 1 Australian Health Workforce Institute, University of Melbourne, Parkville Vic 3010, Australia

## 

Australia has one of the best health systems in the world on many parameters but is facing great challenges with an ageing population increasingly effected with chronic disease. There is also an unrealistic expectation amongst the community as to what the health system can provide and at what cost. A major challenge is the workforce – since it too is ageing. What is important as we move forward is asking questions such as what health services we want to provide, who should provide those services and where, how are those health professionals to be trained and how are they going to be funded. Health services in the future will be increasingly delivered by teams – with different members of those teams performing various tasks – working in a loose delegation model and with all members trained to competency standards for that particular task. Routine tasks – including procedures now only carried out by medical practitioners may well be delegated to appropriately trained others – again working as part of that team. Current providers who wish to participate in delivering these advanced services will need to be appropriately trained and may well be required to work in teams rather than independently. Training will provide a standard of care delivered by the health professional that produces similar (if not better) clinical outcomes to that being provided at present .In this regard interprofessional learning and simulation should feature high on the educational agenda .Dialogue needs to occur between the various professional groups to see how the change agenda can be progressed . Current entrenched positions by existing professional groups need to be addressed in the context of patient care and equity and access of provision of service rather than professional ‘turf ‘. New models of care need to be established, tested in the environment in which they will be used, and if successful then allowed to be implemented with all of the legislative and financial changes required to ensure appropriate uptake in the community.

